# Highly efficient *in vitro* biosynthesis of silver nanoparticles using *Lysinibacillus sphaericus* MR-1 and their characterization

**DOI:** 10.1088/1468-6996/16/1/015004

**Published:** 2015-01-30

**Authors:** Yujun Gou, Rongying Zhou, Xiujuan Ye, Shanshan Gao, Xiangqian Li

**Affiliations:** 1 Faculty of Life Science and Chemical Engineering, HuaiYin Institute of Technology, Huaian 223003, People’s Republic of China; 2 Jiangsu Provincial Engineering Laboratory for Biomass Conversion and Process Integration, Huaian 223003, People’s Republic of China

**Keywords:** silver nanoparticles, green synthesis, *Lysinibacillus sphaericus*, biomaterials, characterizations

## Abstract

Silver nanoparticles (AgNPs) have been widely used in diverse fields due to their superior properties. Currently the biosynthesis of AgNPs is in the limelight of modern nanotechnology because of its green properties. However, relatively low yield and inefficiency diminish the prospect of applying these biosynthesized AgNPs. In this work, a rapid mass AgNP biosynthesis method using the cell-free extract of a novel bacterial strain, *Lysinibacillus sphaericus* MR-1, which has been isolated from a chemical fertilizer plant, is reported. In addition, the optimum synthesis conditions of AgNPs were investigated. The optimum pH, temperature, dosage, and reaction time were 12, 70 °C, 20 mM AgNO_3_, and 75 min, respectively. Finally, AgNPs were characterized by optical absorption spectroscopy, zeta potential and size distribution analysis, x-ray diffraction, electron microscopy, and energy-dispersive x-ray spectroscopy. The results revealed that these biosynthesized AgNPs were bimolecular covered, stable, well-dispersed face centered cubic (fcc) spherical crystalline particles with diameters in the range 5–20 nm. The advantages of this approach are its simplicity, high efficiency, and eco-friendly and cost-effective features.

## 1. Introduction

Over the last few decades, silver nanoparticles (AgNPs) have received substantial attention due to their attractive electronic, chemical, and optical properties [1, 2], and they have been widely used in many fields, including catalysis, optical sensing, and electronics [3, 4]. Currently multi-drug resistance is a growing problem in the treatment of infectious diseases. AgNPs have been applied to disinfection and therapeutics, such as infected burn and wound reduction, medical device sterilization, tumor therapy, and cardiovascular implants [5, 6]. AgNPs are also incorporated into daily-life products, such as apparel, cosmetics, and plastics because of their antimicrobial properties [7, 8]. The antimicrobial activity of AgNPs makes them an excellent choice for multiple uses in the medical field.

AgNPs are generally synthesized by physical and chemical methods [9]. Although these two methods have been able to efficiently produce large quantities of AgNPs with a defined size and shape, they are expensive and often involve the use of toxic and hazardous chemicals, which pose an environmental risk [10]. Furthermore, other problems have generally been associated with these two conventional synthetic routes, such as the aggregation of AgNPs [11, 12]. Therefore, there is growing awareness of the need to develop environmentally friendly and sustainable methods. Microbial synthesis of nanoparticles is a green chemistry approach that interconnects nanotechnology and microbial biotechnology. Following the initial report on the formation of AgNPs in *Pseudomonas stutzeri* [13], many reports on the synthesis of AgNPs using fungi or bacteria have appeared in the literature [14]. Using microorganisms, especially their cell-free extracts, for the synthesis of AgNPs can be advantageous compared with other biological processes because microbial resources are abundant in nature, are easy to culture, and have the potential to be scaled up for large-scale synthesis. However, the biosynthesis of AgNPs by microorganisms has usually involved the use of a low concentration of Ag^+^(i.e., 1 mM) and a long reaction time (on the order of hours or days), which have been two major obstacles to rapid production [15]. Syed and coworkers have reported the reduction of 1 mM of AgNO_3_ into AgNPs within 96 h via the mycelia of the thermophilic fungus *Humicola* sp. [16]. Qian *et al* have investigated the assisted synthesis of AgNPs in 24 h via the cell filtrate of the endophytic fungus *Epicoccum nigrum* by using 1 mM of AgNO_3_ [17]. Also, Malhotra and coauthors have revealed the biosynthesis of AgNPs in 16 h using 1 mM of AgNO_3_ via the cell-free supernatant of a novel marine strain of *Stenotrophomonas* [18]. Consequently, for the purpose of commercial use, the inefficiency and low yield of AgNP biosynthesis need to be overcome urgently.

In this study, a novel approach to the highly efficient and rapid biosynthesis of metallic AgNPs using the cell-free extract of a novel bacterial strain, *Lysinibacillus sphaericus* MR-1, was investigated. The cell-free extract of *L. sphaericus* MR-1 reduced the high concentration of Ag^+^ into AgNPs within several tens of minutes through the use of 20 mM of AgNO_3_. The biosynthesized AgNPs were characterized by various techniques such as ultraviolet-visible absorption spectroscopy (UV–vis), Fourier transform infrared spectroscopy (FTIR), x-ray diffraction (XRD), field-emission scanning electron microscopy (FESEM) combined with energy-dispersive x-ray spectroscopy (EDX), and high-resolution transmission electron microscopy (HRTEM).

## 2. Material and methods

### 2.1. Strain, medium, and chemicals

The strain was isolated from soil samples collected from a chemical fertilizer plant in Huaian by the dilution plate technique on a nutrient agar plate (1% peptone, 0.3% beef extract, 0.5% NaCl, 2% agar, pH 7.5) at 37 °C. It was identified by 16 S rRNA gene sequencing performed by Sangon Biotech (Shanghai) Co., Ltd A yeast extract, peptone, and KNO_3_ (YPK) medium (pH 7.5), which consisted of 0.15% yeast extract, 0.25% peptone, and 0.1% KNO_3_, was employed to culture the isolate. AgNO_3_ was purchased from Sinopharm Chemical Reagent Co., Ltd All the reagents were of analytical grade and were used as purchased without any further purification.

### 2.2. Cell-free extract preparation

For inoculum preparation, a single clone of the isolate was transferred from the nutrient agar plate into 100 ml of sterile nutrient broth in a 250 ml Erlenmeyer flask. The flask was incubated at 37 °C for 12 h on a rotary shaker at 200 rpm. Then 10 ml of mid-log phase culture (optical density at 600 nm OD600 = 1, monitored by UV–vis spectrophotometer) were inoculated into 1000 ml Erlenmeyer flasks containing 500 ml of YPK medium. The inoculated flasks were incubated at 37 °C and shaken at 200 rpm again for 36 h, and then the cell-free extract was obtained by centrifugation (10 000 rpm, 10 min at room temperature) and decantation.

### 2.3. Biosynthesis of AgNPs

For the biosynthesis of the AgNPs, 0.085 g of solid AgNO_3_ was added into 100 ml of cell-free extract in 250 ml Erlenmeyer flasks. The reaction was carried out at 45 °C overnight in the dark on the rotary shakers. The YPK medium with the solid AgNO_3_ and the cell-free extract without the addition of the AgNO_3_ were treated under the same conditions as the controls. The visual color change in the reaction mixture from light yellow to dark brown was observed overnight with reference to the controls. The formation of silver nanoparticles was confirmed by UV–vis spectrophotoscopy.

### 2.4. Optimization of the AgNP synthesis conditions

The effect of three variables (substrate concentration, pH, and temperature) on the production of AgNPs was optimized by varying one parameter at a time, such as the substrate concentration (1–20 mM solid AgNO_3_), pH (6, 7, 8, 8.5, 9, 10, 11, 12, and 13), and temperature (20, 30, 35, 40, 45, 50, 60, 70, 80, and 90 °C). After biosynthesis, 1 ml of the AgNP mixture was taken out and centrifuged, and the pellets were washed three times. Then the pellets were re-suspended in 1 ml of deionized water with pipettes, and the solution was diluted with 10 folders and characterized with UV–vis. Under optimal conditions, the efficiency of the AgNP synthesis was evaluated by characterizing the samples made at different time intervals (0, 15, 30, 45, 60, 75, 90, 120, 180, and 240 min).

### 2.5. Characterization of AgNPs

The AgNP mixture was centrifuged at 10 000 rpm for 30 min to isolate the AgNPs from free proteins or other compounds present in the solution, following which the pellets were re-dispersed in sterile distilled water to get rid of any uncoordinated biological molecules. The process of centrifugation and re-dispersion in sterile distilled water was repeated three times to ensure better separation of free entities from the mixture. The localized surface plasmon resonance (SPR) of the AgNPs was characterized with a UV–vis spectrophotometer (UV-2401PC, Shimadzu, Japan) at a resolution of 1 nm in a wavelength range between 300 and 600 nm. The hydrodynamic diameter and the zeta potential of the AgNPs were measured by dynamic light scattering (DLS) using a Malvern Zetasizer Nano ZS 90 (Worcestershire, UK). HRTEM images were recorded using a Tecnai G2 F30 S-TWIN microscope operated at an accelerating voltage of 200 kV. Samples for HRTEM imaging were prepared by placing a drop of the solution sample in deionized water onto a carbon-coated Cu grid, drying it in air, and loading it into the electron microscope chamber.

Then the purified pellets were freeze-dried, and the powders were subjected to FTIR and XRD measurement. The Fourier transform infrared spectra were recorded by a Nicolet 5700, Thermo Electron Co., USA, in the range 400–4000 cm^−1^ at a resolution of 4 cm^−1^. X-ray powder diffraction measurements were carried out on a Bruker D8 Advance (Germany) instrument in Bragg–Brentano mode with Cu K*α* radiation from 35 to 90° 2*θ*. These purified solid AgNPs were further ultrasonically dispersed with ethanol, dropped on glass slides, air dried, and examined by FESEM combined with EDX (Quanta 250, USA). The numerical data of these characterizations were processed by the software package Origin Pro 8.5.

## 3. Results

### 3.1. Morphology and molecular analysis of the isolate

**Figure 1.**
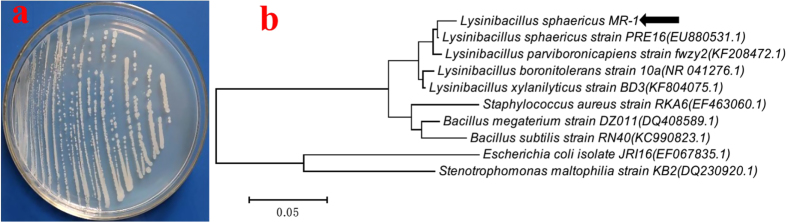
(a) Pure pale-white colonies of *L. sphaericus* MR-1 isolated from soil by a nutrient agar plate. (b) Phylogenetic tree constructed through the neighbor-joining method based on 16 S rRNA gene nucleotide sequences of *L. sphaericus* MR-1 and a reference sequence retrieved from NCBI Gen Bank.

The pure pale-white bacterial X11 (figure 1(a)) obtained on the nutrient agar plate was identified as *L. sphaericus* based on molecular analysis through 16 S rRNA sequencing studies (figure 1(b)) and was named *L*. *sphaericus* MR-1.

### 3.2. Biosynthesis of AgNPs

**Figure 2.**
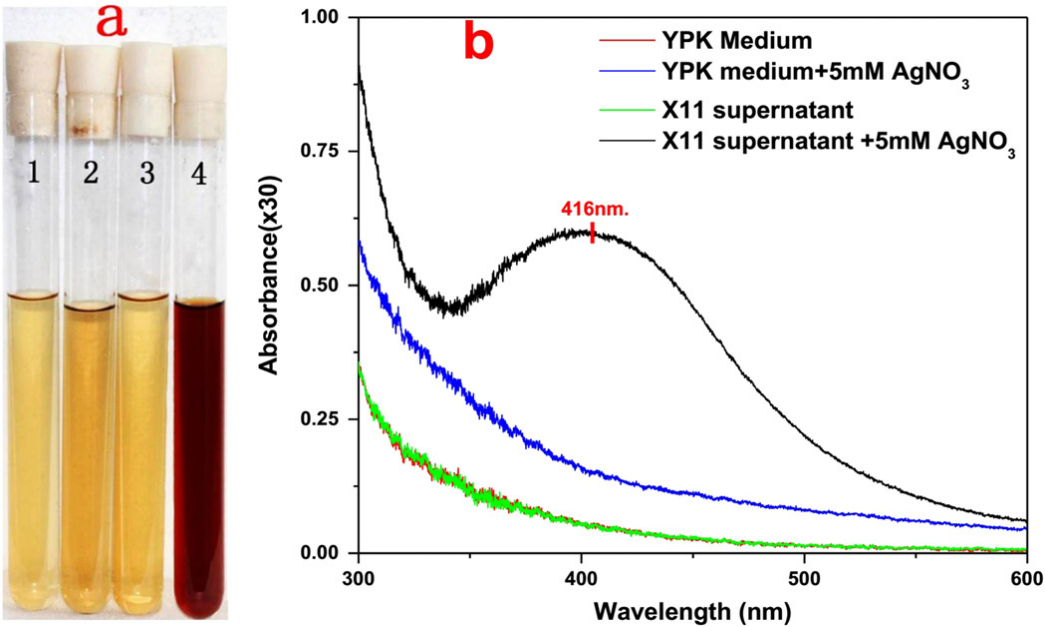
(a) Color change of reaction mixture containing different constituents (1. YPK medium; 2. YPK medium with 5 mM of AgNO_3_; 3. *L*. *sphaericus* MR-1 (X11) cell-free extract; 4. *L. sphaericus* MR-1 (X11) cell-free extract with 5 mM AgNO_3_). After overnight incubation, only the *L. sphaericus* MR-1 (X11) cell-free extract with 5 mM of AgNO_3_ showed an obvious color change from clear pale yellow to dark brown, confirming the formation of silver nanoparticles. (b) UV–vis spectrum of the reaction mixture containing different constituents, only the *L*. *sphaericus* MR-1 (X11) cell-free extract after the overnight incubation of the 5 mM of AgNO_3_ showed a peak at 416 nm, further confirming the formation of silver nanoparticles.

In the experiment, the formation of AgNPs was visually confirmed by the color change of the mixture from pale yellow to dark brown. This change did not occur in the negative controls (figure 2(a)). The preliminary investigation of the biosynthesized AgNPs was carried out by UV–vis spectroscopic analysis. It can be readily observed that the characteristic surface plasmon resonance band peak of the mixture was at 416 nm (figure 2(b)), indicating the presence of AgNPs [19].

### 3.3. Optimization of the AgNP synthesis conditions

**Figure 3.**
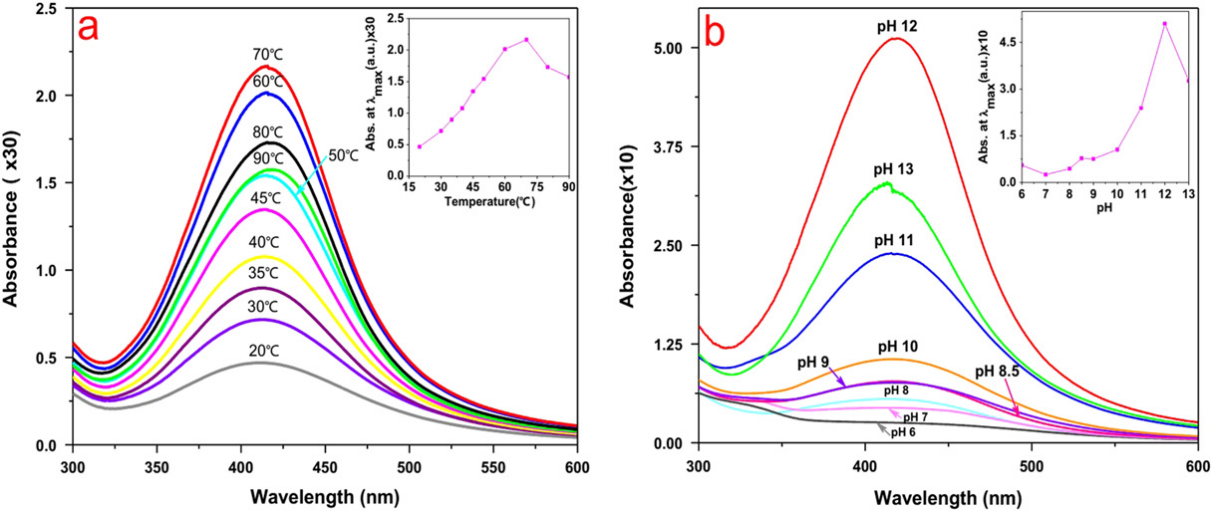
(a) Effect of temperature on silver nanoparticle synthesis; inset shows a nearly linear relationship between maximum absorbance and a temperature in the range 20–70 °C. (b) Effect of pH on AgNP synthesis by *L*. *sphaericus* MR-1 cell-free extract, which showed that alkaline pH was a necessary condition of this biosynthesis procedure and pH 12 was an optimum condition; the inset shows the relationship between the maximum absorbance and pH in the range 6–13.

While the effect of temperature on the synthesis of AgNPs was being investigated, it was found that the maximum absorbance of the reaction mixture had increased from 20 to 70 °C, whereas it had decreased from 70 to 90 °C. As shown in the inset of figure 3(a), a nearly linear relationship between the maximum absorbance and the temperature in the range 20–70 °C was presented. The result suggested that moderately elevated temperature accelerated the reduction process.

In general, the reduction reaction of metallic ions is sensitive to the pH of a solution [20, 21]. The current study involved a systematic analysis of pH-dependent changes in the reaction mixture for the synthesis of AgNPs. It was observed that the maximum absorbance had increased when pH increased from 6 to 12 (figure 3(b)). The result indicated that an alkaline pH favored the formation of AgNPs.

**Figure 4.**
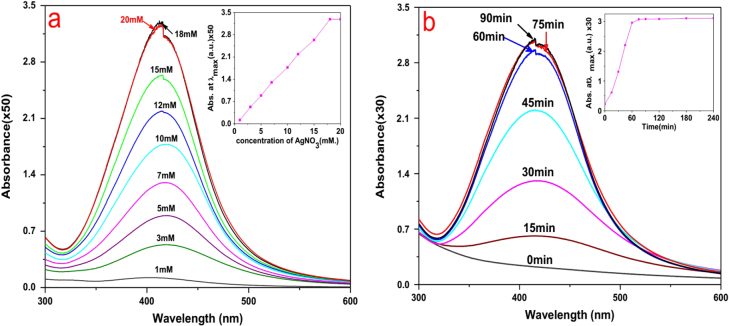
(a) Effect of concentration of AgNO_3_ on AgNP synthesis by *L*. *sphaericus* MR-1 cell-free extract, which showed that 18–20 mM was an optimum condition; the inset shows the relationship between the maximum absorbance and a concentration of AgNO_3_ in the range 1–20 mM. (b) Time evolution of AgNP synthesis under the optimal condition; the inset shows the linear relationship between the maximum absorbance and the reduction time.

In addition, we evaluated the effect of different concentrations of AgNO_3_ on the synthesis of AgNPs. The maximum synthesis of AgNPs occurred with respect to Ag^+^ concentration in the range 18–20 mM (figure 4(a)), and a few earlier researchers also showed that optimum AgNP accumulation occurred under this condition [22].

The UV–vis spectrum of AgNP synthesis under optimal conditions as a function of time is shown in figure 4(b). After 45, 60, and 75 min, the reduction was 71.5%, 96%, and 100% completed, respectively. It can be seen that the reduction was quite rapid and was much faster than previously reported bioreduction processes, which were on the order of hours or days, and was comparable to or faster than many chemical or physical methods [15, 23].

### 3.4. Characterization of AgNPs

**Figure 5.**
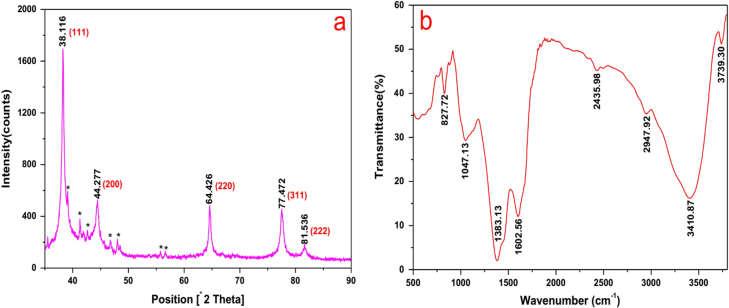
(a) X-ray diffraction pattern of silver nanoparticles synthesized by cell-free extract of *L. sphaericus* MR-1, which showed the 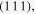 (2 0 0), (2 2 0), (3 1 1), and (222) crystallographic planes of face centered cubic (fcc) AgNPs. The small peaks marked with (∗) may originate from the AgCl or Ag_2_O crystals in the sample. (b) FTIR spectrum of silver nanoparticles synthesized by cell-free extract of *L*. *sphaericus* MR-1.

The typical XRD pattern of the biosynthesized AgNPs is shown in figure 5(a). Five diffraction peaks at 2*θ* values of 38.116°, 44.277°, 64.426°, 77.472°, and 81.536°, corresponding to the d-spacing values 2.359, 2.044, 1.445, 1.231, and 1.180 Å of the AgNPs, were observed. They were assigned to the (1 1 1), (2 0 0), (2 2 0), (3 1 1), and (2 2 2) crystalline planes of the face centered cubic (fcc) crystalline structure of metallic silver, respectively (JCPDS file no. 00-004-0783). The broad nature of the XRD peaks could be attributed to the nanocrystalline nature of the AgNPs. The small peaks appeared to have possibly originated from the AgCl or Ag_2_O crystals in the sample. The surface chemistry of the biosynthesized AgNPs was investigated using FTIR spectroscopy (figure 5(b)). The peaks seen at 3739.3, 3410.9, 2947.9, 2436.0, 1602.6, and 1383.1 cm^−1^ were assigned to the O–H, N–H, –O–CN, –CN, C=C, and C–O groups, respectively [24–26]. The two bands observed at 1047.1 and 827.7 cm^−1^ can be attributed to the –O– stretching vibrations of aromatic and aliphatic amines [27, 28]. These functional groups may have an effective role in the green synthesis of AgNPs. The overall result confirmed the presence of bimolecular in the sample. It is reported that proteins can bind to nanoparticles through either free amine groups or cysteine residues in the proteins [29]. Therefore, reduction and stabilization of the AgNPs by proteins in the cell-free extract may have occurred in this procedure.

**Figure 6.**
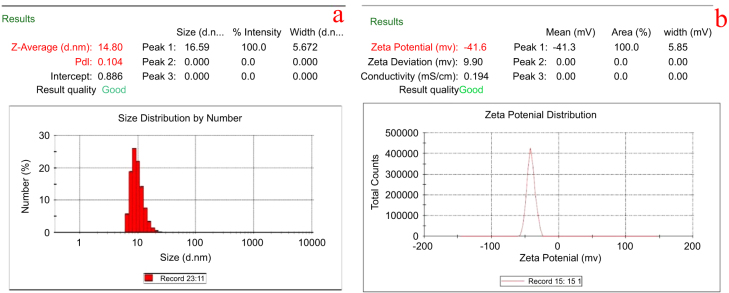
Dynamic light scattering measurements for (a) particle size distribution analysis and (b) zeta potential measurements of AgNPs.

The average size distribution of silver nanoparticles in colloidal solution was found to be 14.8 ± 1.2 nm (figure 6(a)). A negative zeta potential of about −41.6 ± 0.5 mV was observed in the current study that represents the ideal surface charge (figure 6(b)). A high absolute value of zeta potential denotes a high electrical charge on the surface of AgNPs, which can cause a strong repulsive force among the particles to prevent agglomeration and which thus might be responsible for the stable nature of the AgNPs.

**Figure 7.**
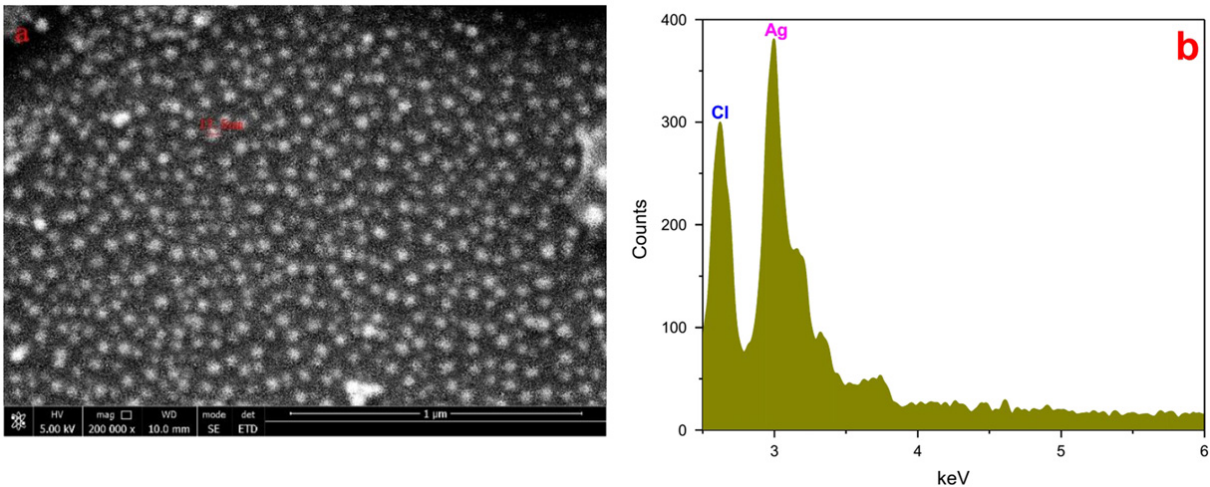
(a) FESEM image of silver nanoparticles, which were 5–20 nm in size, spherical, and well dispersed. (b) EDX spectrum of samples recorded in the area-profile mode, which clearly shows the Ag signals.

**Figure 8.**
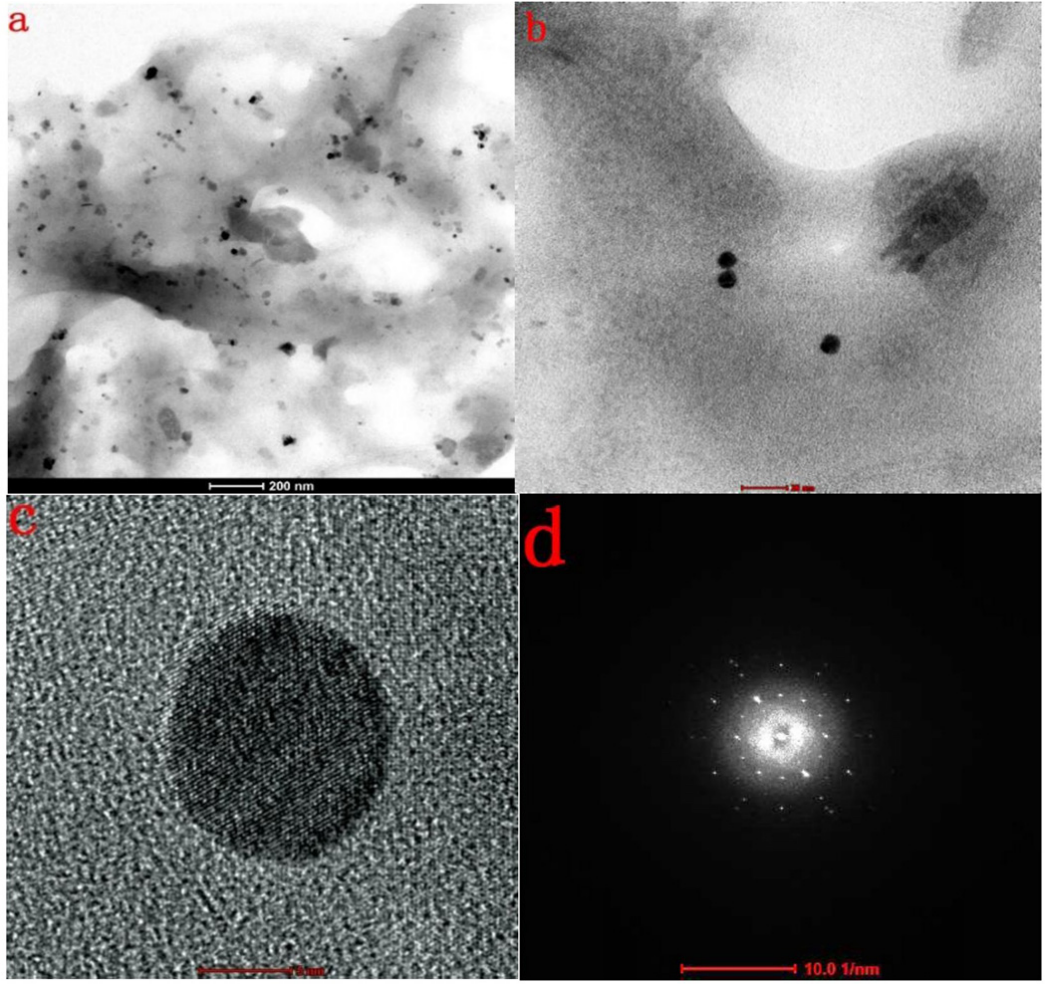
(a) Overview TEM image of AgNPs biosynthesized using the cell-free extract of *L. sphaericus* MR-1, showing that the particles are well dispersed (scale bar: 200 nm). (b) Typical TEM image of as-biosynthesized AgNPs (scale bar: 20 nm). (c) Typical HRTEM image of a single AgNP (scale bar: 5 nm). (d) typical selected area electron diffraction pattern of AgNPs biosynthesized using the cell-free extract of *L. sphaericus* MR-1 (scale bar: 10 1/nm).

An FESEM image of the AgNPs synthesized under optimum conditions using 20 mM of silver nitrate solution revealed that the particles were spherical and well dispersed and had diameters of 5–20 nm (figure 7(a)). The EDX spectrum (figure 7(b)) confirmed the formation of AgNPs. The presence of Cl may have come from the glass slides used for the EDX sample preparation. The HRTEM images (figure 8) further demonstrated the nature of AgNPs. The particle diameter was around 5–10 nm. The size seemed a little smaller than that revealed by the FESEM image and size distribution analysis, which might be explained as a result of the bimolecular coating on the AgNPs.

## 4. Discussion

Biogenic silver nanoparticles are an interesting alternative to chemically and physically produced AgNPs due to their green properties. But to be commercially available and able to compete with chemically and physically synthesized AgNPs, the production of biogenic AgNPs needs to be improved. This can be achieved by carefully choosing biological resources such as plants and microorganisms. Some plants have been used to produce AgNPs with high yield. For example, Maria *et al* used the extract of the stem bark of *Z. xylopyrus* obtained by the reflex extraction method to synthesize AgNPs. Under the condition of pH 11, 28 ± 2 °C, and 10 mM AgNO_3_, the extract reduced about 60% of the AgNO_3_ into 60–70 nm AgNPs after 24 h [30]. However, large-scale production of biogenic AgNPs by the microbial method has not been explored so far. In this paper, the cell-free extract of *Lysinibacillus sphaericus* MR-1 was used to synthesize AgNPs. Under the condition of pH 12, 70 °C, and 20 mM of AgNO_3_, the extract reduced AgNO_3_ into 5–20 nm AgNPs in 75 min. Since the reducing agent (cell-free extract of *L. sphaericus* MR-1) can be easily obtained through fermentation and this microorganism resource is a renewable green material, this method for biosynthesis of AgNPs can be suitably scaled up for large-scale commercial synthesis.

In his review Berry concluded that *L*. *sphaericus* bacteria were usually used as an insect pathogen because of their parasporal crystal (BT) endotoxins [31]. Also, Fayaz *et al* investigated the synergistic effect of biosynthesized AgNPs and antibiotics [32]. Since AgNPs have a significant antimicrobial effect, the discovery of the considerable capability of this strain to synthesize AgNPs may open the door to developing multifunctional agriculture products. For example, a product functions as an insecticide and at the same time an anti-pathogen. Liong *et al* and Otsuka *et al* used modified silver nanoparticles synthesized by a chemical method as drug carriers [33, 34]. Since the AgNPs synthesized by the cell-free extract of *L*. *sphaericus* MR-1 were covered by bimolecular, such AgNPs can be used as drug carriers in the field of biomedicine because of the functional groups on their surface.

## 5. Conclusions

In this study, we report rapid mass biosynthesis of AgNPs with the features of simplicity, high reduction rate, and high yield. A novel bacterial strain, *L*. *sphaericus* MR-1, was isolated from the soil of a chemical fertilizer plant. The cell-free extract of the *L*. *sphaericus* MR-1 reduced a high concentration of Ag^+^ to AgNPs rapidly with high yield. Under the condition pH 12, 70 °C, and 20 mM of AgNO_3_, the reduction was completed within 75 min. These AgNPs were characterized by various techniques, including UV–vis, XRD, FTIR, FESEM-EDX, HRTEM, zeta potential, and size distribution analysis. Results revealed that these AgNPs were monodispersed, stable, spherical in the range of 5–20 nm, and coated with biological molecules. The molecules covering the surface provide ample room for functionalized modifications, which indicates that AgNPs possess significant potential for application in the field of biomedicine.
